# Supplementary figures and images for: Regulatory changes in the fatty acid elongase eloF underlie the evolution of sex-specific pheromone profiles in Drosophila prolongata
Source: BMC Biol. 2025 Apr 30;23:117. doi: 10.1186/s12915-025-02220-z (PMC12044895; doi:10.1186/s12915-025-02220-z)

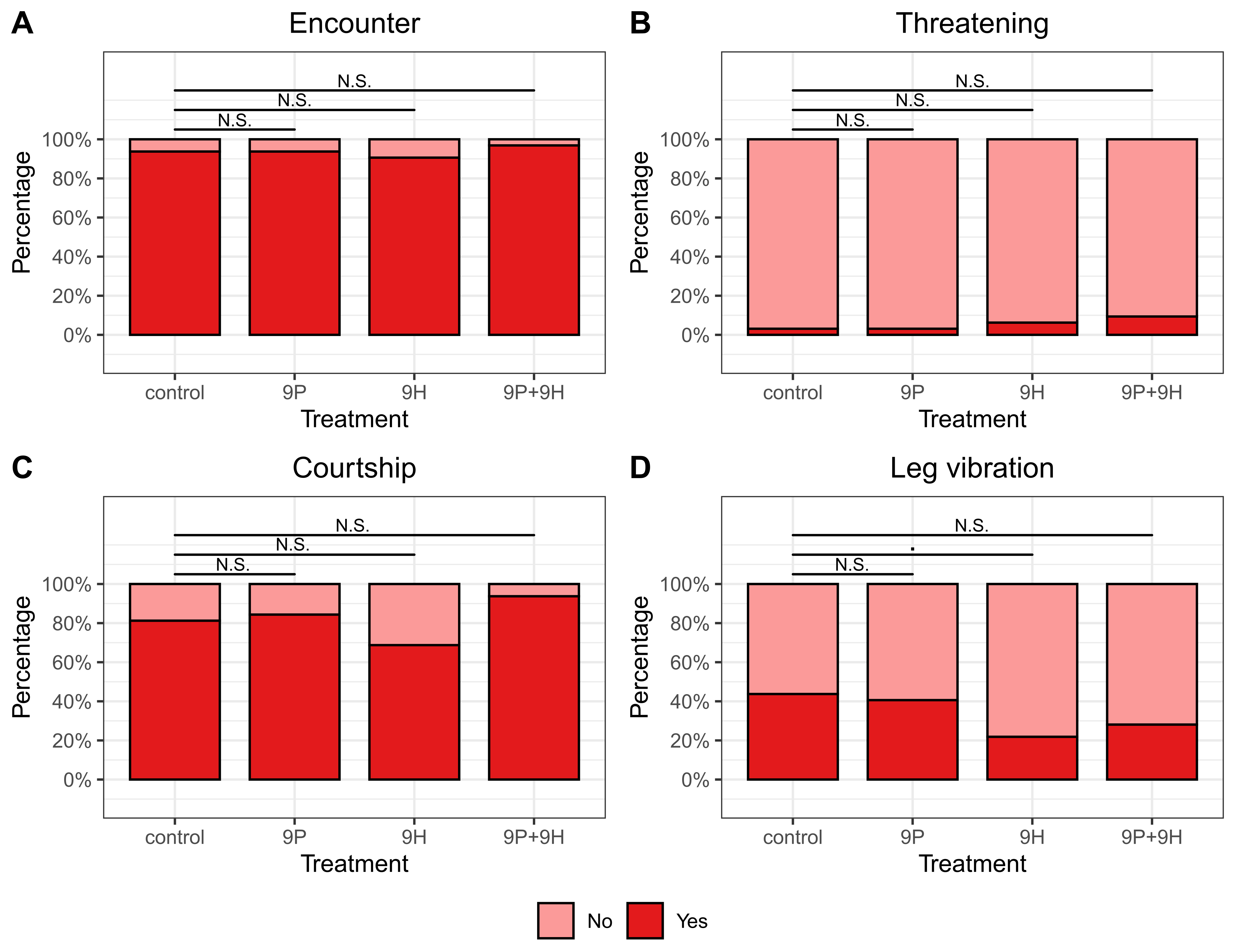

Supplement: Supplementary file 1 — Additional file 1: Figure S1. Variable effect of perfuming on male-female interactions. Stacked bar plots showing success rates of (A) Encounter, (B) Threatening, (C) Courtship (defined as the proportion of males that continued to court the female after the initial encounter), and (D) Leg vibration (where the male vigorously shakes the female’s abdomen with his front legs) across three perfuming conditions (N = 32 for each treatment). N.S., nonsignificant results based on comparison between treatment and control in a logistic regression model. P values are as follows: *** p < 0.001, ** p < 0.01, *, p < 0.05, p < 0.1. [file 12915_2025_2220_MOESM1_ESM.tif]

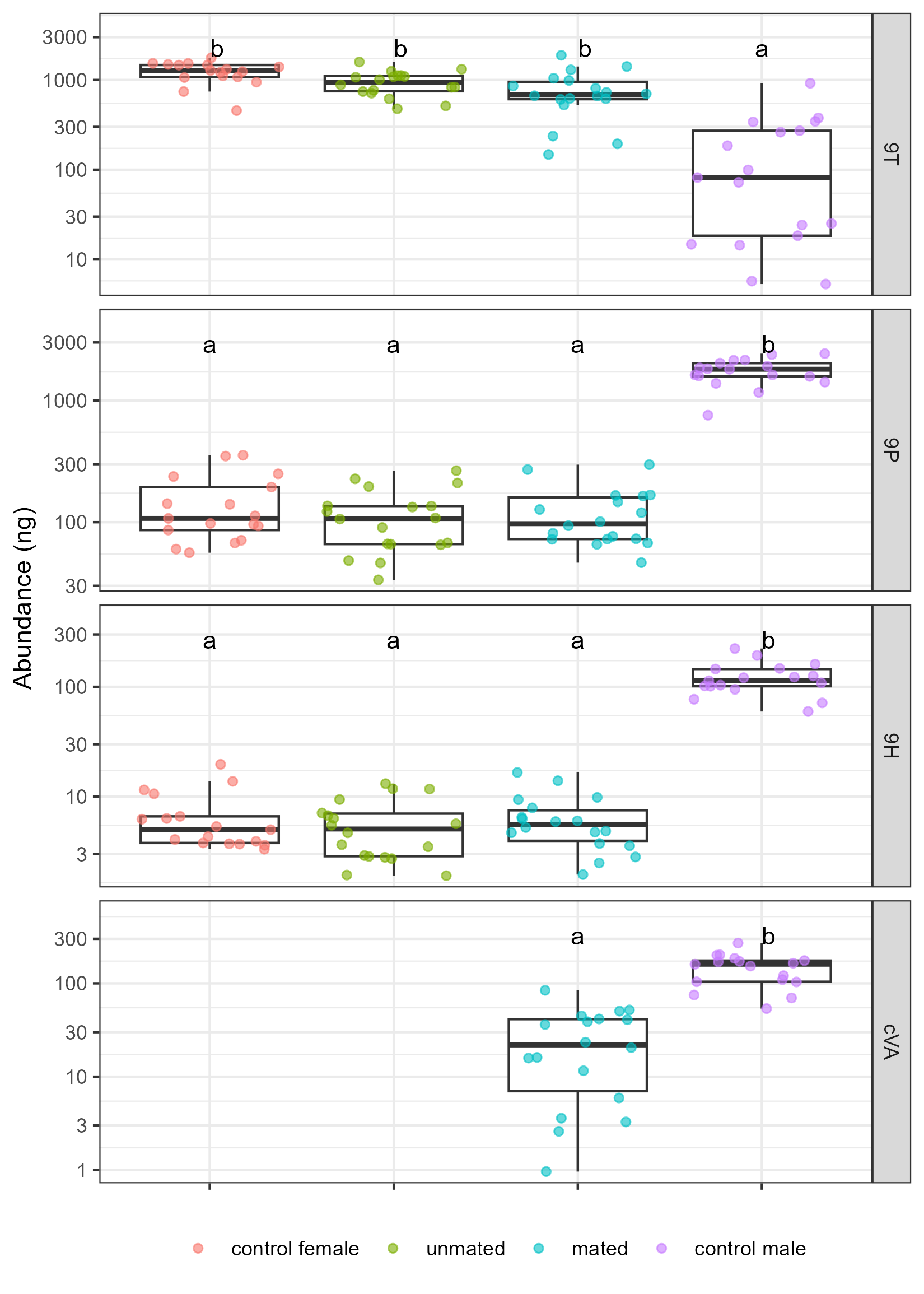

Supplement: Supplementary file 2 — Additional file 2: Figure S2. Male-biased long-chain CHCs are not transferred to females during mating. Boxplots showing the abundance of 9T, 9P and 9H, with cis-vaccenyl-acetate (cVA) as a positive control. Shown are control wild-type females, WT females that did not mate with a WT male, WT females that mated with a WT male, and control WT males. Pheromone abundance is measured in nanograms per fly and shown on log10 scale. Overlayed jitter points are samples of each sex * mating status combination, color-coded by genotype. Significance results of all pairwise comparisons (Tukey HSD test followed by significant omnibus ANOVA F-tests) are summarized in the format of compact letter display (using R packages"multicomp" and "lsmeans"). [file 12915_2025_2220_MOESM2_ESM.tif]

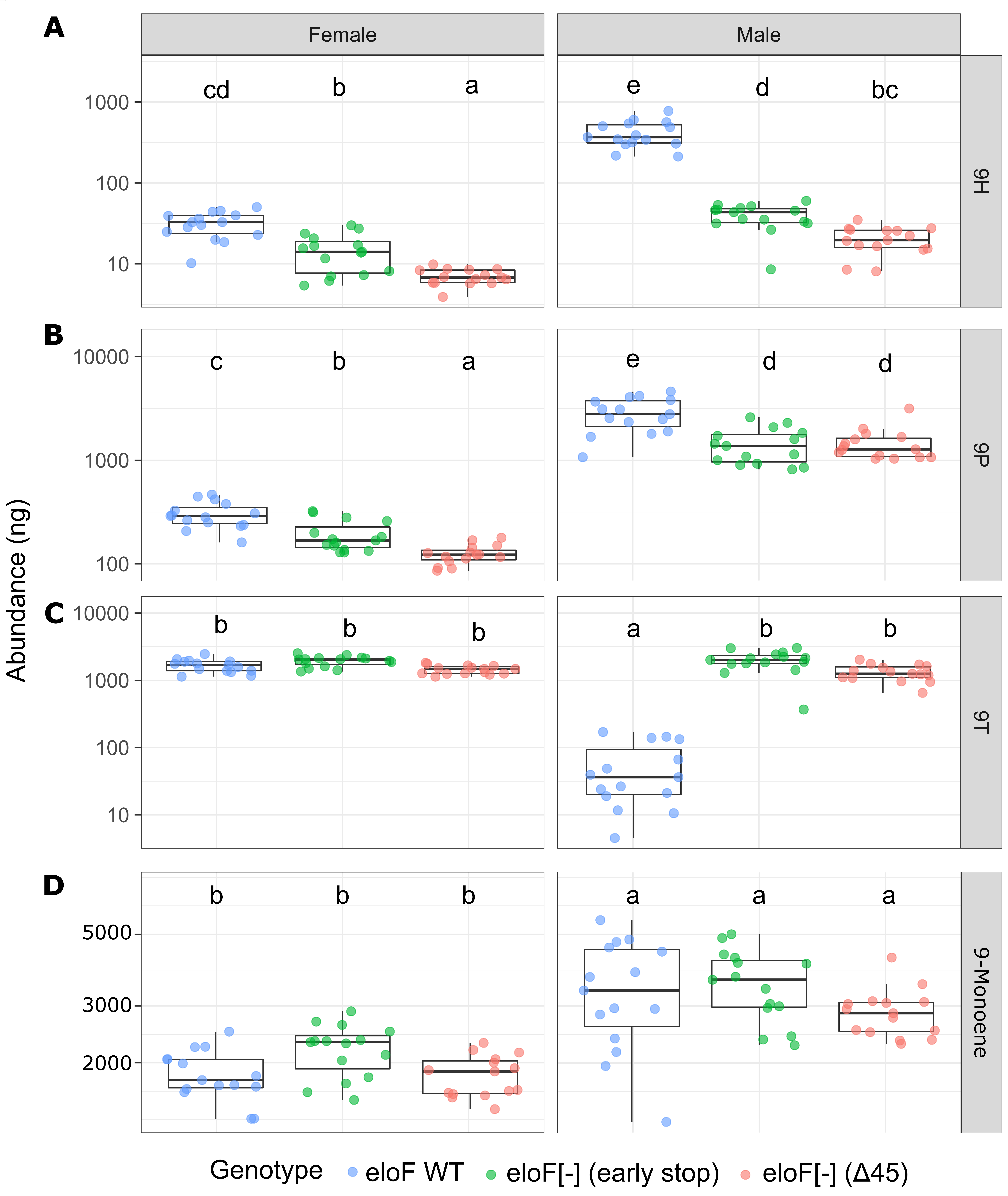

Supplement: Supplementary file 7 — Additional file 7: Figure S5. eloF is responsible for elongating the precursors of long-chain 9-monoenes. Boxplots showing the abundance of 9H (A), 9P (B), 9T (C), and the aggregate 9-Monoenes (D) across genotypes in each sex, with abundance in nanograms shown on log10 scale. Overlayed jitter points are samples of each sex * genotype combination, with color-coded genotypes. Significance of all pairwise comparisons (Tukey HSD test followed by significant omnibus ANOVA F-tests) are summarized in the format of compact letter display (using R packages "multicomp" and "lsmeans"). Note the decrease in the abundance of 9P and 9H in both sexes, and an increase in the abundance of 9T in males, in eloF mutants, while the total abundance of 9-monoenes remains approximately constant. [file 12915_2025_2220_MOESM7_ESM.tif]

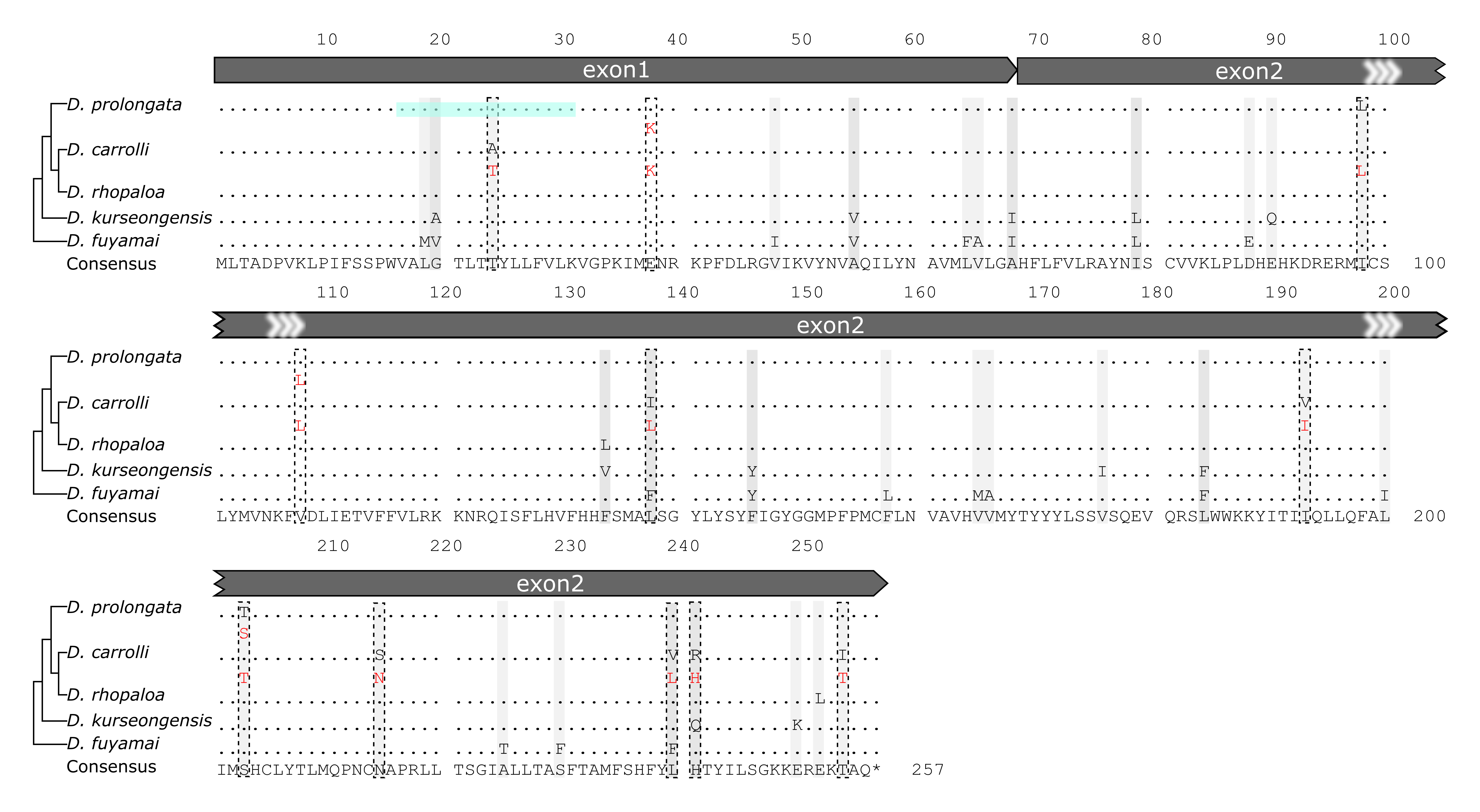

Supplement: Supplementary file 11 — Additional file 11: Figure S8. No fixed protein sequence differences between D. prolongata and D. carrolli eloF orthologs. Multiple alignment on translated amino acid sequences across five species in the rhopaloa species subgroup, with species phylogeny on the left and the consensus sequence at the bottom. Numbers above the consensus sequence are coordinates showing the consensus length (257 AA). For the alleles of each species, site-wise disagreement from the consensus is represented in gray shade. For D. carrolli and D. prolongata, single nucleotide polymorphisms (SNPs) that lead to changes in amino acids are highlighted in red. Polymorphic sites are represented in dashed rectangles. In D. prolongata, amino acid sequences deleted in one CRISPR mutant (eloF[-] Δ45) are in cyan shade. Feature annotations are displayed above the protein sequence, with dark gray boxes representing eloF exons. All features have their direction labeled as arrowheads. [file 12915_2025_2220_MOESM11_ESM.tif]

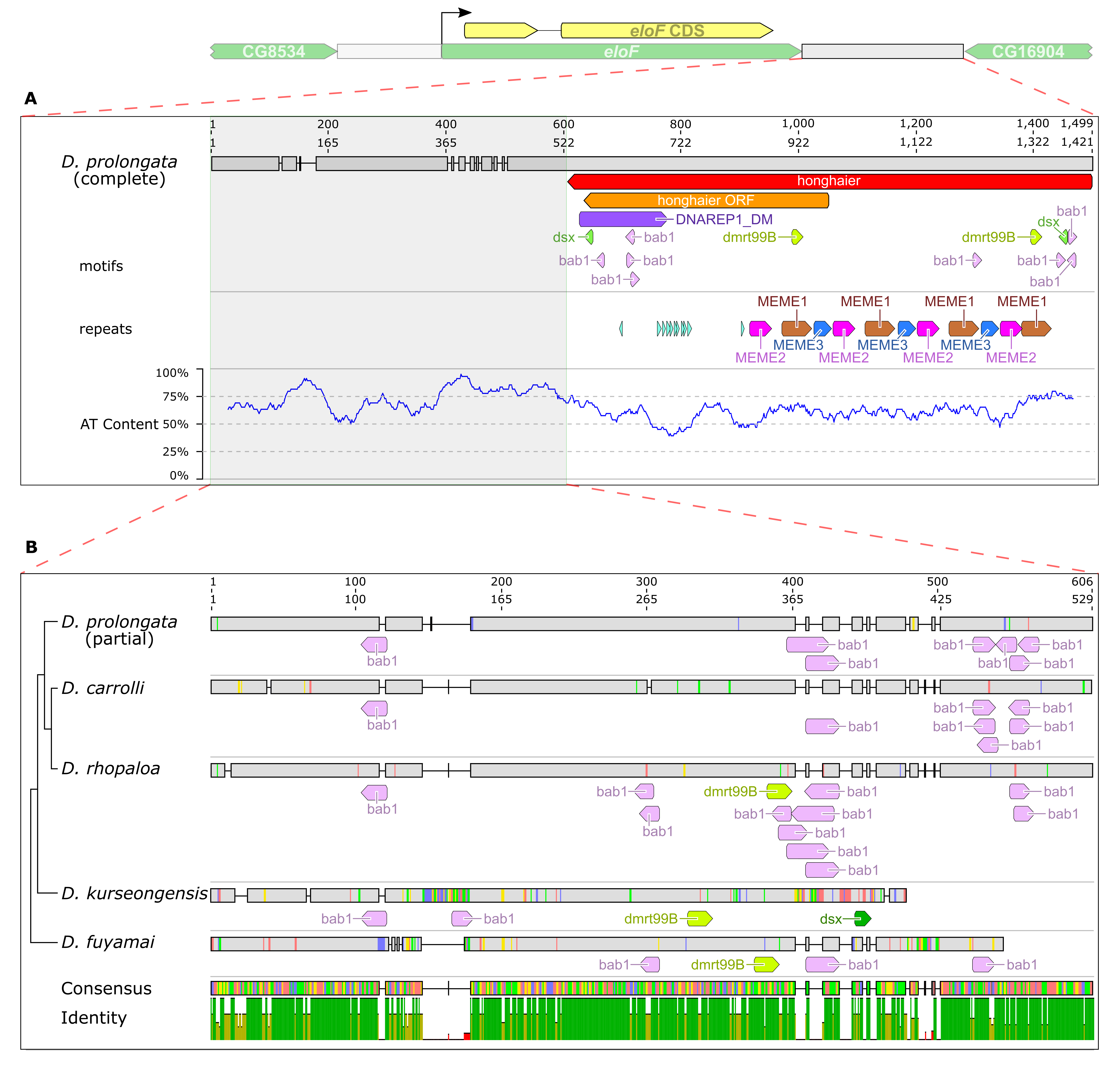

Supplement: Supplementary file 13 — Additional file 13: Figure S9. D. prolongata-specific honghaier insertion in the downstream region of eloF. (A) The downstream region of eloF in D. prolongata, showing the insertion of the TE-like repetitive element honghaier. Feature annotations are displayed below DNA sequence, with the red box representing the honghaier insertion, the orange box representing its predicted ORF, and the purple box showing the BLAST hit to the DNAREP_DM1 transposable element (Dfam). The motif track shows putative binding sites for transcription factors including dsx (JASPAR, dark green), dsx (FlyReg, light green), dmrt99B (JASPAR, yellow-green), and bab1 (iDMMPMM, pink). The repeat track includes short TGTC repeats (cyan) and three de novo motifs: MEME-1 (brown), MEME-2 (pink), and MEME-3 (steel blue). (B) Alignment of the conserved downstream region of eloF (shaded region in A) across species, with species phylogeny on the left and consensus sequence at the bottom. Numbers above the DNA sequence are coordinates showing the length of the consensus (606 bp) and alignment (529 bp). For the alleles of each species, nucleotide-wise disagreement from the consensus is represented in a color-coded vertical line for nucleotide substitutions (A: red, C: blue, G: yellow, T: green), and a horizontal line for nucleotide deletions. The track of percent identity is color coded as follows: green for perfect (100%) agreement, yellow-green for intermediate (30-99%) agreement, and red for low (<30%) agreement. All features have their direction labeled as arrowheads when applicable. [file 12915_2025_2220_MOESM13_ESM.tif]

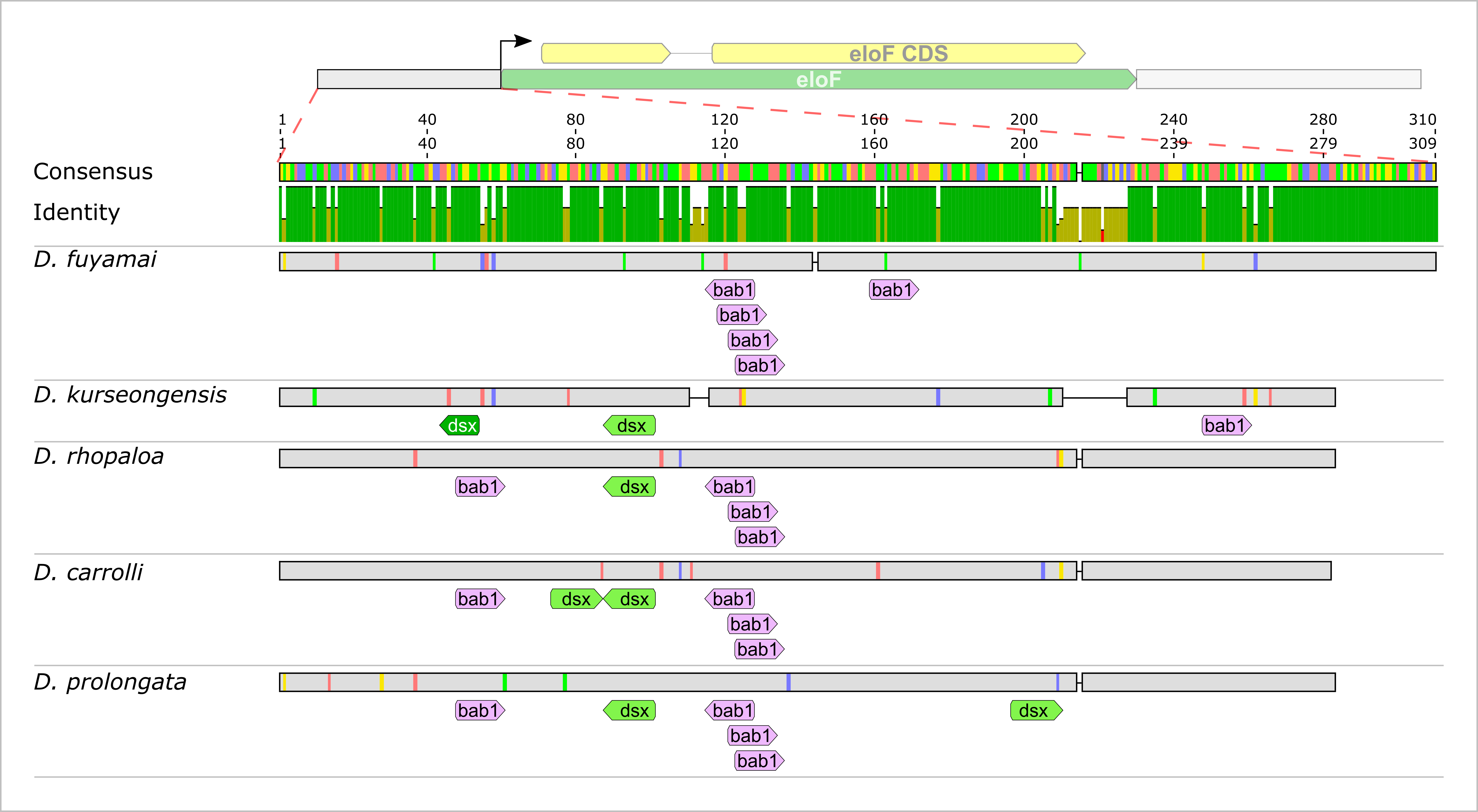

Supplement: Supplementary file 15 — Additional file 15: Figure S10. The upstream region of eloF is conserved in the rhopaloa species subgroup. Multiple alignment of the upstream region of eloF, with schematic gene structure displayed on top. The track of percent identity is color-coded as follows: green for perfect (100%) agreement, yellow-green for intermediate (30-99%) agreement, and red for low (<30%) agreement. Numbers above the percent identity track are coordinates showing the length of the consensus (310 bp) and alignment (309 bp). For alleles from each species, nucleotide-wise disagreement from the consensus is represented in a color-coded vertical line for nucleotide substitutions (A: red, C: blue, G: yellow, T: green), and a horizontal line for nucleotide deletions. Predicted transcription factor (TF) binding motifs are displayed below the DNA sequence as follows: dsx (JASPAR, dark green); dsx (FlyReg, light green); bab1 (iDMMPMM, pink). All features have their direction labeled as arrowheads when applicable. [file 12915_2025_2220_MOESM15_ESM.tif]

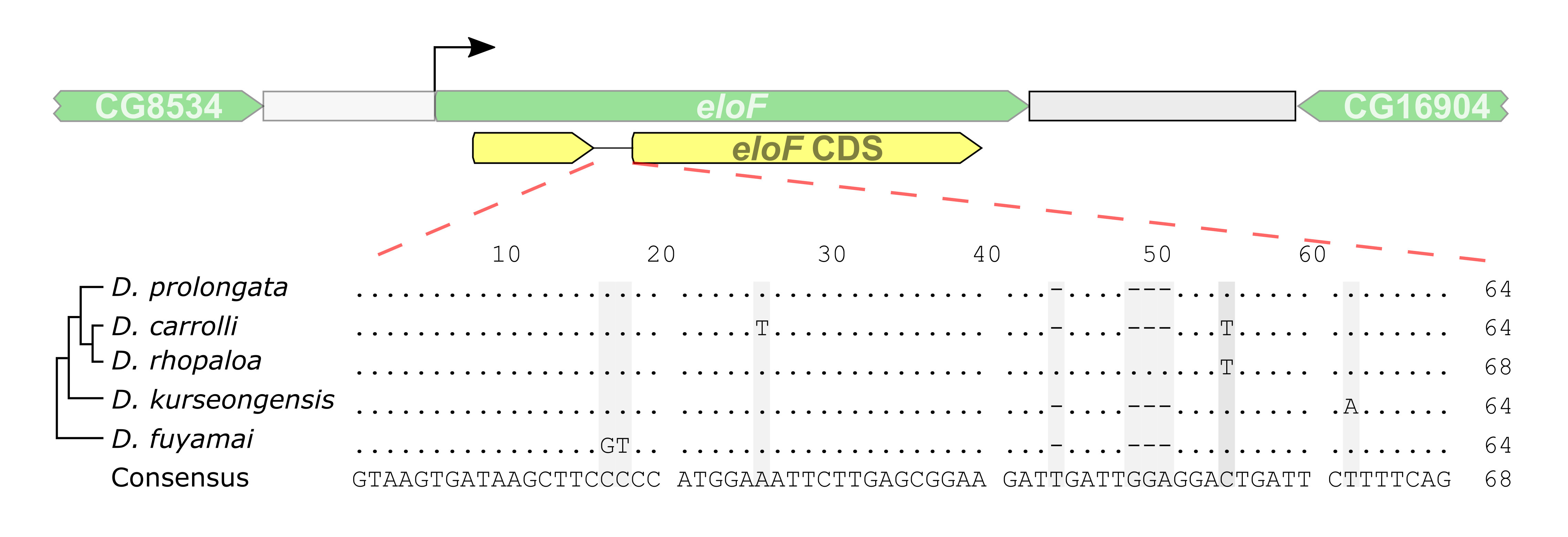

Supplement: Supplementary file 16 — Additional file 16: Figure S11. The intron of eloF in conserved in the rhopaloa species subgroup. Multiple alignment of the intronic region of eloF, with genomic context displayed on top. Numbers above the DNA sequence are coordinates showing the consensus length (68 bp). For the alleles of each species, site-wise disagreement from the consensus is represented in gray shade. No sex (dsx) or tissue (bab1) motifs were identified. [file 12915_2025_2220_MOESM16_ESM.tif]

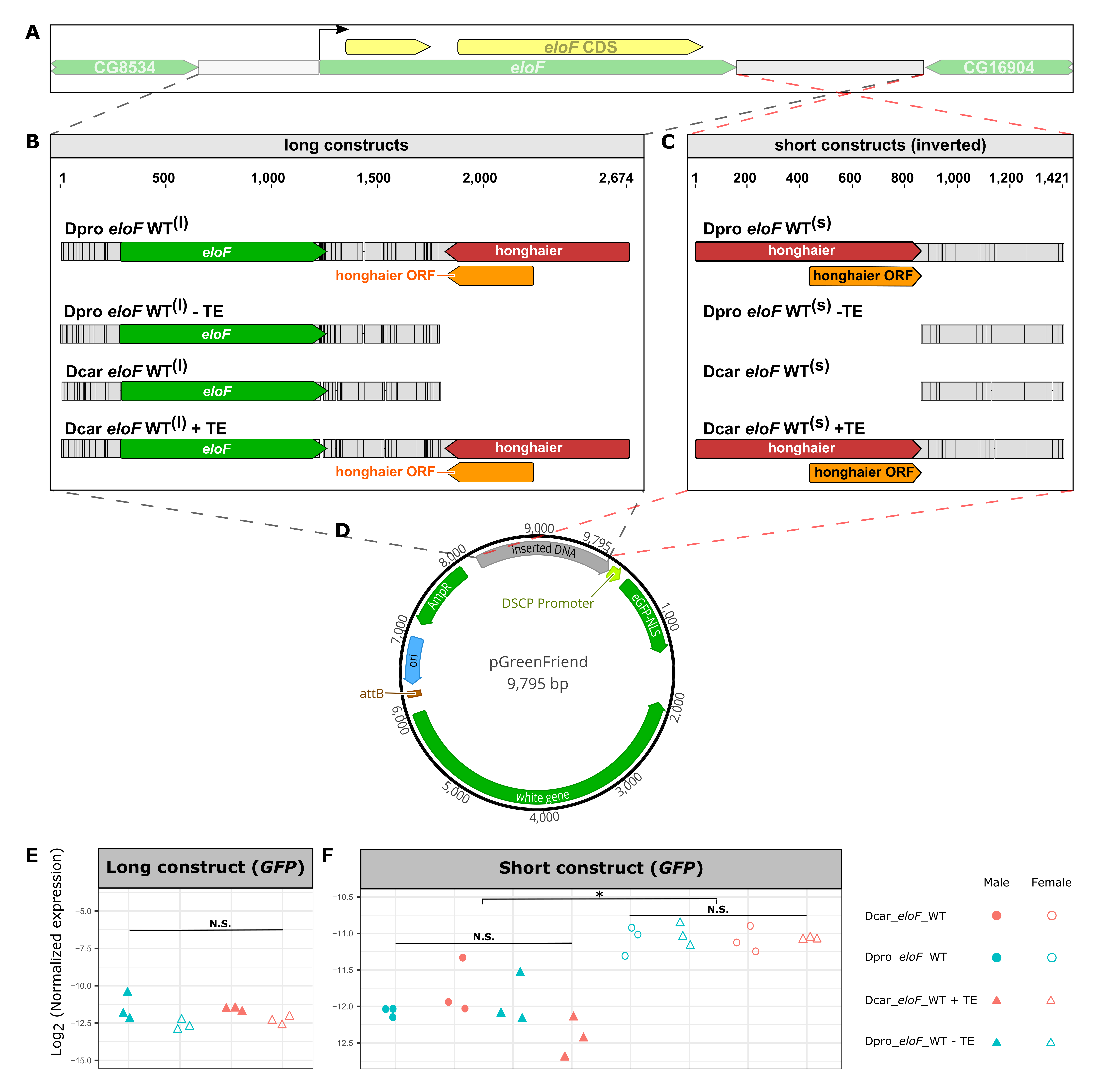

Supplement: Supplementary file 17 — Additional file 17: Figure S12. Design and analysis of GFP reporter constructs containing eloF sequences. (A) Schematic illustration of the eloF locus and the two flanking genes. (B) “Long” constructs containing the entire eloF locus including flanking sequences. Dpro eloF WT(l) and Dcar eloF WT(l) carry wild-type eloF loci from D. prolongata and D. carrolli, respectively. The other two constructs were made by removing the honghaier TE insertion from the D. prolongata sequence (Dpro eloF WT(l)-TE) or adding the D. prolongata honghaier insertion to the D. carrolli sequence (Dcar eloF WT(l)+TE). The eloF locus is placed into the pGreenFriend vector in the forward orientation, so that eloF is transcribed in the same direction as GFP while the honghaier insertion is in the opposite direction. (C).“Short” constructs containing only the downstream eloF sequences. As in the “long” constructs, two constructs contain the wild-type alleles from D. prolongata and D. carrolli, while the other two were made by TE swap. Here, the downstream eloF sequences are placed into the pGreenFriend vector in the flipped orientation, so that the direction of the honghaier insertion is the same as GFP transcription. In (B) and (C), alignment coordinates are displayed on top. Black lines indicate disagreement between theD. prolongata and D. carrolli alleles, vertical for single nucleotide variants and horizontal for short indels. Feature annotations are displayed below DNA sequence, with green box representing genes, yellow box representing CDS, red box representing the honghaier insertion, and the orange box representing its predicted ORF. All features have their direction labeled by arrowheads when applicable. (D) Schematic illustration of pGreenFriend vector, where GFP is driven by the Drosophila synthetic core promoter (DSCP, yellow-green). (E) In the “long” constructs containing the entire eloF locus, GFP reporter expression is low and does not differ significantly between genotypes. (F) [file 12915_2025_2220_MOESM17_ESM.tif]

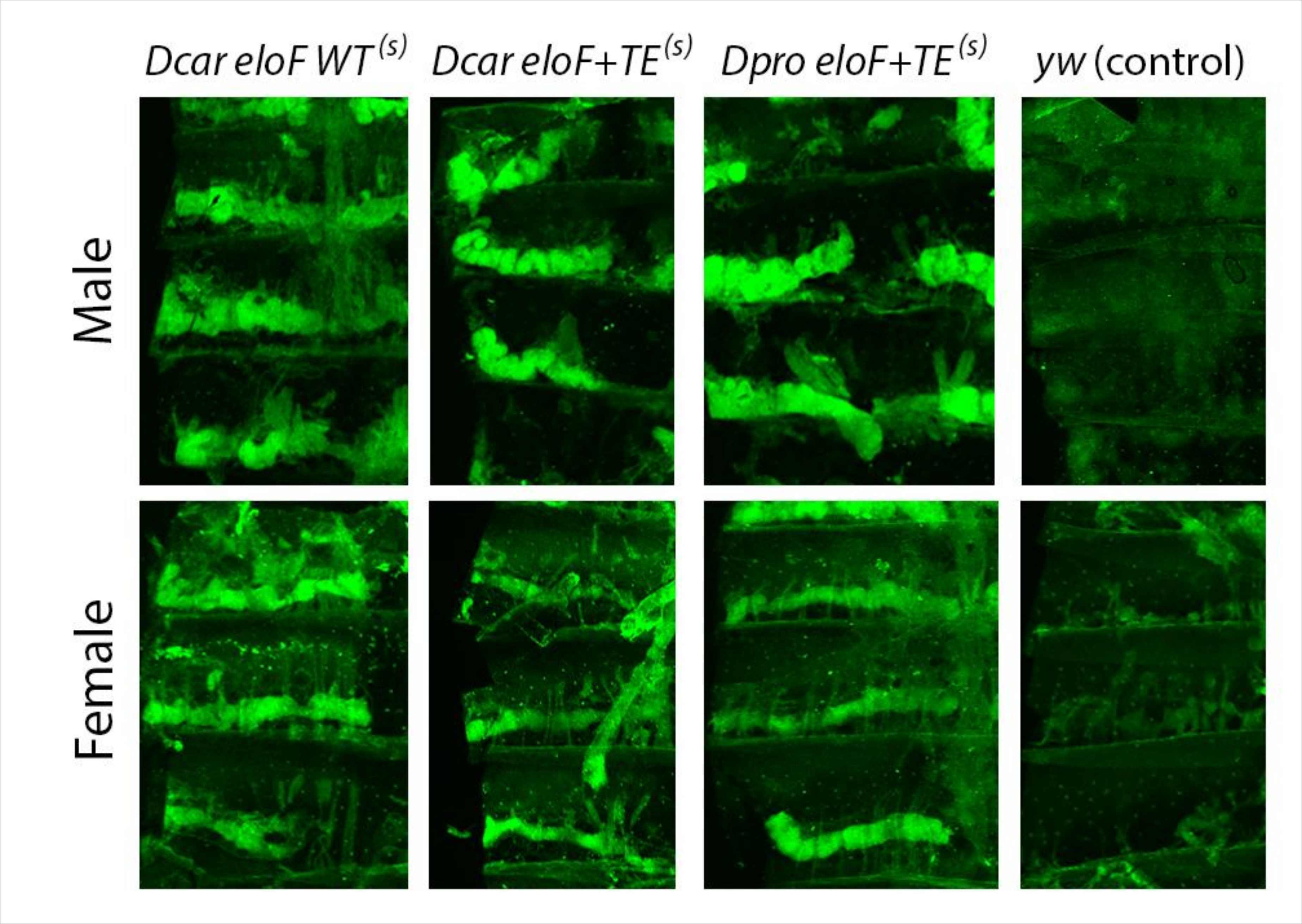

Supplement: Supplementary file 19 — Additional file 19: Figure S13. eloF downstream sequences drive GFP expression in adult abdominal oenocytes. Confocal images of GFP protein stained with anti-GFP antibodies, showing dissected male and female dorsal abdominal body walls. Non-transgenic yw flies are used as a negative control. Transgenic flies carry the “short” constructs containing the eloF downstream region (see Additional file 17: Fig S12). [file 12915_2025_2220_MOESM19_ESM.tif]

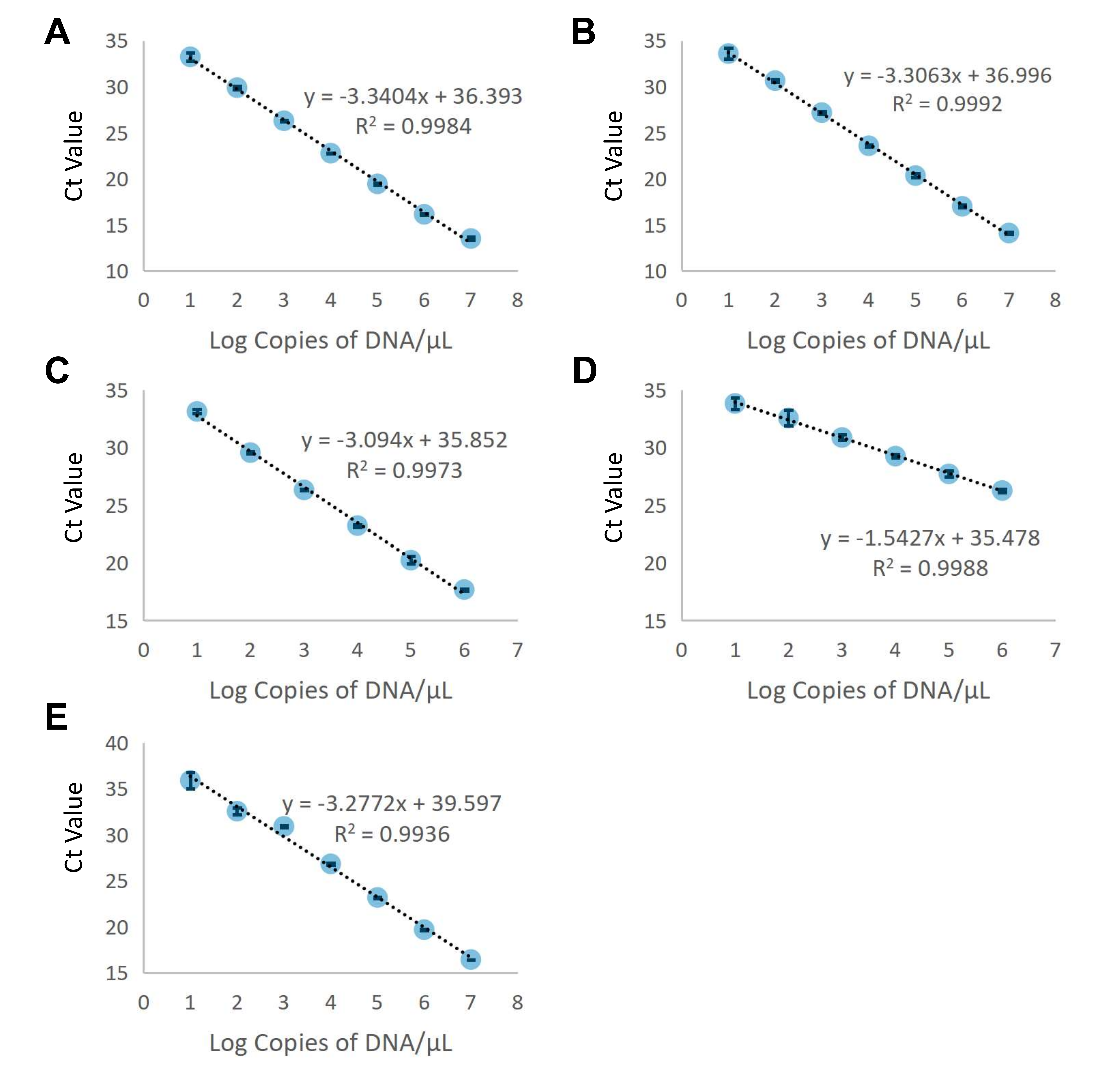

Supplement: Supplementary file 29 — Additional file 29: Figure S14. All primers used for quantitative PCR have near-perfect amplification performance. Standard curves of Rpl32 are based on cDNA from mixed-sex whole-body RNA of D. prolongata (A) and D. carrolli (B). Standard curves of eloF are based on cDNA from mixed-sex whole-body RNA of D. prolongata (C) and D. carrolli (D). Standard curve of GFP is based on empty pGreenFriend vector (E). Dilution factors are 10-fold for (A), (B) and (E); 8-fold for (C), and 3-fold for (D). Points represent average values, with error bars showing standard deviations calculated from three technical replicates. Lines represent the best linear fit, showing the estimated equation and coefficient of determination (R2). [file 12915_2025_2220_MOESM29_ESM.tif]
