# Supplementary material for: Regulatory changes in the fatty acid elongase eloF underlie the evolution of sex-specific pheromone profiles in Drosophila prolongata
Source: BMC Biol. 2025 Apr 30;23:117. doi: 10.1186/s12915-025-02220-z (PMC12044895; doi:10.1186/s12915-025-02220-z)
Supplement: Supplementary file 4 — Additional file 4: Table S1. Significant GO terms in the comparison between males of D. prolongata and D. carrolli. [file 12915_2025_2220_MOESM4_ESM.docx]

Table S1. Significant GO terms in the comparison between males of *D. prolongata* and *D. carrolli*.

| GO.ID | Term | Annotated | Significant | Expected | Fisher | KS | Rank in KS | Rank in Fisher | Mean rank |
| --- | --- | --- | --- | --- | --- | --- | --- | --- | --- |
| GO:0006749 | glutathione metabolic process | 26 | 17 | 7.43 | 1.00E-04 | 3.00E-04 | 2 | 1 | 1.5 |
| GO:0030148 | sphingolipid biosynthetic process | 29 | 17 | 8.29 | 0.00067 | 0.0047 | 9 | 2 | 5.5 |
| GO:0032504 | multicellular organism reproduction | 649 | 163 | 185.43 | 0.00171 | 0.0014 | 4 | 3 | 3.5 |
| GO:0009064 | glutamine family amino acid metabolic process | 15 | 10 | 4.29 | 0.00241 | 0.0036 | 8 | 4 | 6 |
| GO:0006487 | protein N-linked glycosylation | 22 | 13 | 6.29 | 0.00263 | 0.0076 | 14 | 5 | 9.5 |
| GO:0009063 | cellular amino acid catabolic process | 33 | 17 | 9.43 | 0.00442 | 5.00E-04 | 3 | 6 | 4.5 |
| GO:0035167 | larval lymph gland hemopoiesis | 41 | 15 | 11.71 | 0.00459 | 0.0143 | 28 | 7 | 17.5 |
| GO:0042761 | very long-chain fatty acid biosynthetic process | 14 | 9 | 4 | 0.00575 | 0.0138 | 27 | 8 | 17.5 |
| GO:0061077 | chaperone-mediated protein folding | 35 | 13 | 10 | 0.00581 | 0.0079 | 16 | 9 | 12.5 |
| GO:0007442 | hindgut morphogenesis | 28 | 9 | 8 | 0.00668 | 0.0147 | 29 | 10 | 19.5 |
| GO:0002118 | aggressive behavior | 10 | 7 | 2.86 | 0.00785 | 0.0062 | 12 | 11 | 11.5 |
| GO:0006720 | isoprenoid metabolic process | 18 | 9 | 5.14 | 0.00865 | 0.0097 | 19 | 12 | 15.5 |
| GO:0006081 | cellular aldehyde metabolic process | 17 | 10 | 4.86 | 0.00868 | 0.0028 | 7 | 13 | 10 |
| GO:0007186 | G protein-coupled receptor signaling pathway | 61 | 25 | 17.43 | 0.00881 | 0.0212 | 38 | 14 | 26 |
| GO:0022409 | positive regulation of cell-cell adhesion | 13 | 8 | 3.71 | 0.0133 | 0.0274 | 44 | 16 | 30 |
| GO:0007552 | metamorphosis | 331 | 89 | 94.57 | 0.01397 | 0.0121 | 24 | 17 | 20.5 |
| GO:0044248 | cellular catabolic process | 618 | 182 | 176.57 | 0.01423 | 0.0238 | 40 | 18 | 29 |
| GO:0006641 | triglyceride metabolic process | 29 | 13 | 8.29 | 0.01461 | 0.0265 | 42 | 19 | 30.5 |
| GO:0007166 | cell surface receptor signaling pathway | 440 | 111 | 125.71 | 0.01534 | 0.0102 | 20 | 20 | 20 |
| GO:0006730 | one-carbon metabolic process | 11 | 7 | 3.14 | 0.0163 | 0.0019 | 5 | 22 | 13.5 |
| GO:0019367 | fatty acid elongation, saturated fatty acid | 11 | 7 | 3.14 | 0.0163 | 0.0291 | 49 | 23 | 36 |
| GO:0034625 | fatty acid elongation, monounsaturated fatty acid | 11 | 7 | 3.14 | 0.0163 | 0.0291 | 50 | 24 | 37 |
| GO:0034626 | fatty acid elongation, polyunsaturated fatty acid | 11 | 7 | 3.14 | 0.0163 | 0.0291 | 51 | 25 | 38 |
| GO:0034446 | substrate adhesion-dependent cell spreading | 11 | 7 | 3.14 | 0.0163 | 0.0333 | 56 | 26 | 41 |
| GO:0035336 | long-chain fatty-acyl-CoA metabolic process | 11 | 7 | 3.14 | 0.0163 | 0.0394 | 65 | 27 | 46 |
| GO:0008045 | motor neuron axon guidance | 34 | 16 | 9.71 | 0.01655 | 0.0127 | 25 | 28 | 26.5 |
| GO:0044282 | small molecule catabolic process | 113 | 44 | 32.29 | 0.01741 | 0.0187 | 36 | 29 | 32.5 |
| GO:0015849 | organic acid transport | 37 | 15 | 10.57 | 0.02317 | 0.0083 | 18 | 31 | 24.5 |
| GO:0044782 | cilium organization | 31 | 12 | 8.86 | 0.02323 | 0.0181 | 35 | 32 | 33.5 |
| GO:0006605 | protein targeting | 88 | 28 | 25.14 | 0.02325 | 0.0418 | 68 | 33 | 50.5 |
| GO:0000578 | embryonic axis specification | 76 | 18 | 21.71 | 0.0236 | 0.0283 | 48 | 34 | 41 |
| GO:0031333 | negative regulation of protein-containing complex assembly | 21 | 8 | 6 | 0.02568 | 0.0282 | 47 | 36 | 41.5 |
| GO:1901606 | alpha-amino acid catabolic process | 25 | 12 | 7.14 | 0.03047 | 0.0021 | 6 | 41 | 23.5 |
| GO:0055085 | transmembrane transport | 283 | 94 | 80.86 | 0.03356 | 0.0341 | 59 | 42 | 50.5 |
| GO:0046112 | nucleobase biosynthetic process | 10 | 6 | 2.86 | 0.03749 | 0.0351 | 60 | 43 | 51.5 |
| GO:0090407 | organophosphate biosynthetic process | 140 | 39 | 40 | 0.03853 | 0.0059 | 11 | 47 | 29 |
| GO:1901607 | alpha-amino acid biosynthetic process | 26 | 12 | 7.43 | 0.04225 | 0.0369 | 64 | 48 | 56 |
| GO:0016042 | lipid catabolic process | 74 | 27 | 21.14 | 0.04811 | 0.0403 | 67 | 50 | 58.5 |
| GO:0007472 | wing disc morphogenesis | 213 | 58 | 60.86 | 0.04826 | 0.0271 | 43 | 51 | 47 |

Fisher: raw p-values from Fisher’s exact test

KS: raw p-values from the Kolmogorov-Smirnov test
