# Supplementary material for: Regulatory changes in the fatty acid elongase eloF underlie the evolution of sex-specific pheromone profiles in Drosophila prolongata
Source: BMC Biol. 2025 Apr 30;23:117. doi: 10.1186/s12915-025-02220-z (PMC12044895; doi:10.1186/s12915-025-02220-z)
Supplement: Supplementary file 6 — Additional file 6: Table S2. Cuticular lipid description. [file 12915_2025_2220_MOESM6_ESM.docx]

Table S2. qPCR analysis of GFP transcript expression driven by *eloF* “long” constructs (complete *eloF* locus including flanking regions).

| Sex | Genotype |  | Reference gene (*Rpl32*) | Gene of interest (*GFP*) |
| --- | --- | --- | --- | --- |
| Female | Dpro *eloF* WT^(l)^ | Replicate 1 | 14.81070235 | >40 |
|  |  | Replicate 2 | 14.46329199 | >40 |
|  |  | Replicate 3 | 14.51088284 | >40 |
|  |  | NRT control |  | >40 |
|  | Dcar *eloF* WT^(l)^ | Replicate 1 | 14.79372247 | >40 |
|  |  | Replicate 2 | 14.66467155 | >40 |
|  |  | Replicate 3 | 14.37498109 | >40 |
|  |  | NRT control |  | >40 |
| Male | Dpro *eloF* WT^(l)^ | Replicate 1 | 13.84065353 | 37.68637158 |
|  |  | Replicate 2 | 14.91291474 | >40 |
|  |  | Replicate 3 | 13.9237942 | 39.51370016 |
|  |  | NRT control |  | >40 |
|  | Dcar *eloF* WT^(l)^ | Replicate 1 | 14.06775107 | 35.15658614 |
|  |  | Replicate 2 | 13.83850168 | 34.86276857 |
|  |  | Replicate 3 | 13.65032641 | 34.52029242 |
|  |  | NRT control |  | >40 |

Note: qPCR amplification Ct values are reported, where non-detects are labeled as >40.
