# Supplementary material for: Regulatory changes in the fatty acid elongase eloF underlie the evolution of sex-specific pheromone profiles in Drosophila prolongata
Source: BMC Biol. 2025 Apr 30;23:117. doi: 10.1186/s12915-025-02220-z (PMC12044895; doi:10.1186/s12915-025-02220-z)
Supplement: Supplementary file 10 — Additional file 10: Table S3. eloF[-] mutant behavior. [file 12915_2025_2220_MOESM10_ESM.docx]

Table S3. Cuticular lipid description

| Compound name | Abbreviation | Chain length | Chemical class | isConsensus | Kovat's index | Characteristic ions (m/z) |
| --- | --- | --- | --- | --- | --- | --- |
| 9-Heneicosene | 9Hen | 21 | 9-Monoene | FALSE | NA | 294 |
| 7-Heneicosene | 7Hen | 21 | 7-Monoene | FALSE | NA | 294 |
| n-Heneicosane | nC21 | 21 | Straight-chain alkane | TRUE | 2100 | 296 |
| 9-Docosene | 9D | 22 | 9-Monoene | FALSE | 2173~2180 | 308 |
| 11-cis-vaccenyl acetate | cVA | 20 | Acetate ester | FALSE | 2190~2191 | 250, 310 |
| n-Docosane | nC22 | 22 | Straight-chain alkane | TRUE | 2200 | 310 |
| 2-Methyl-docosane | 23Br | 23 | Branched alkane | TRUE | 2263~2264 | 281, 309, 324 |
| x,y-Tricosadiene | xyTD | 23 | Diene | FALSE | 2270~2271 | 320 |
| 9-Tricosene | 9T | 23 | 9-Monoene | TRUE | 2274~2278 | 322 |
| 7-Tricosene | 7T | 23 | 7-Monoene | TRUE | 2280~2282 | 322 |
| n-Tricosane | nC23 | 23 | Straight-chain alkane | TRUE | 2300 | 324 |
| 2-Methyl-tricosane | 24Br | 24 | Branched alkane | FALSE | 2363~2364 | 295, 323, 338 |
| 9-Tetracosene | 9Te | 24 | 9-Monoene | TRUE | 2374~2376 | 336 |
| 7-Tetracosene | 7Te | 24 | 7-Monoene | TRUE | 2377~2379 | 336 |
| n-Tetracosane | nC24 | 24 | Straight-chain alkane | TRUE | 2400 | 338 |
| 2-Methyl-tetracosane | 25Br | 25 | Branched alkane | TRUE | 2463~2464 | 309, 337, 352 |
| 9-Pentacosene | 9P | 25 | 9-Monoene | TRUE | 2475~2478 | 350 |
| 7-Pentacosene | 7P | 25 | 7-Monoene | TRUE | 2482~2484 | 350 |
| n-Pentacosane | nC25 | 25 | Straight-chain alkane | TRUE | 2500 | 352 |
| 9-Hexacosene | 9He | 26 | 9-Monoene | FALSE | 2574~2582 | 364 |
| 2-Methyl-hexacosane | 27Br | 27 | Branched alkane | TRUE | 2663~2664 | 337, 365, 380 |
| 9-Heptacosene | 9H | 27 | 9-Monoene | TRUE | 2676~2677 | 378 |
| 7-Heptacosene | 7H | 27 | 7-Monoene | TRUE | 2683~2685 | 378 |
| n-Heptacosane | nC27 | 27 | Straight-chain alkane | TRUE | 2700 | 380 |
| 2-Methyl-octacosane | 29Br | 29 | Branched alkane | TRUE | 2859~2861 | 365, 393, 408 |
| n-Nonacosane | nC29 | 29 | Straight-chain alkane | FALSE | 2900 | 408 |
