# Supplementary material for: Regulatory changes in the fatty acid elongase eloF underlie the evolution of sex-specific pheromone profiles in Drosophila prolongata
Source: BMC Biol. 2025 Apr 30;23:117. doi: 10.1186/s12915-025-02220-z (PMC12044895; doi:10.1186/s12915-025-02220-z)
Supplement: Supplementary file 12 — Additional file 12: Table S4. Sites segregating in the coding region of eloF in D. prolongata and D. carrolli. [file 12915_2025_2220_MOESM12_ESM.docx]

Table S4. *eloF[-]* mutant behavior

|  | Mating behavior | | | | | | Fighting behavior | | | | Misdirected courtship | |
| --- | --- | --- | --- | --- | --- | --- | --- | --- | --- | --- | --- | --- |
| Genotype | N | courtship (rate) | mean courtship duration [min] (SE) | leg vibration  (rate) | copulation  (rate) | mean copulation duration [min] (SE) | N | threatening (rate) | boxing (rate) | mean boxing duration [min] (SE) | N | occurrence (rate) |
| eloF WT | 32 | 21 (0.656) | 3.344 (0.739) | 8 (0.25) | 6 (0.188) | 6.033 (0.506) | 23 | 22 (0.957) | 9 (0.391) | 2.217 (1.016) | 40 | 3 (0.075) |
| eloF[-] (Δ45) | 22 | 12 (0.545) | 7.300 (2.334) | 9 (0.409) | 6 (0.273) | 5.467 (0.436) | 15 | 15 (1.000) | **12 (0.800) *** | 4.913 (1.175) | 40 | 7 (0.175) |
| eloF[-] (early stop) | 26 | 17 (0.654) | 4.754 (1.243) | 4 (0.154) | 2 (0.077) | 5.550 (0.350) | 30 | 27 (0.900) | 10 (0.333) | 2.077 (0.800) | 40 | **13 (0.325) **** |

*eloF* mutations were induced in the reference genome strain (Luecke et al. 2024), which is used here as wild-type control. See Fig 7 for mutant annotations. Behavior was observed for 1 hour. In the mating assay, a single male was paired with a single female of the same genotype. In the fighting assay, two males of the same genotype were placed together. In the misdirected courtship assay, a wild-type male was scored for misdirected courtship (wing vibration, leg vibration or attempted copulation) towards a decapitated male (*eloF[-]* or wild-type) in the presence of a wild-type female. Z-tests were performed on coefficients from logistic regression to determine the p-value for each comparison (eloF[-] against eloF WT), with significant results highlighted in bold. P values are as follows: *** p < 0.001, ** p < 0.01, *, p < 0.05.
