# Supplementary material for: Regulatory changes in the fatty acid elongase eloF underlie the evolution of sex-specific pheromone profiles in Drosophila prolongata
Source: BMC Biol. 2025 Apr 30;23:117. doi: 10.1186/s12915-025-02220-z (PMC12044895; doi:10.1186/s12915-025-02220-z)
Supplement: Supplementary file 14 — Additional file 14: Table S5. High fidelity honghaier sequence occurrence. [file 12915_2025_2220_MOESM14_ESM.docx]

Table S5. Sites segregating in the coding region of *eloF* in *D. prolongata* and *D. carrolli.*

| DNA | | | | | Protein | | | | |
| --- | --- | --- | --- | --- | --- | --- | --- | --- | --- |
| Position | Dpro_Ref | Dpro_Alt | Dcar_Ref | Dcar_Alt | Position | Dpro_Ref | Dpro_Alt | Dcar_Ref | Dcar_Alt |
| 51 | T |  | C | T | 17 | Val |  | Val | Val |
| 69 | A | G | G | A | 23 | Thr | Thr | Thr | Thr |
| 70 | A |  | G | A | 24 | Thr |  | Ala | Thr |
| 90 | C | G | C | G | 30 | Leu | Leu | Leu | Leu |
| 102 | G | C | C | G | 34 | Pro | Pro | Pro | Pro |
| 112 | G | A | G | A | 38 | Glu | Lys | Glu | Lys |
| 135 | G | A | G | A | 45 | Leu | Leu | Leu | Leu |
| 153 | C | G | C | G | 51 | Val | Val | Val | Val |
| 292 | C |  | A | C | 98 | Leu |  | Ile | Leu |
| 322 | G | T | G | T | 108 | Val | Leu | Val | Leu |
| 411 | T |  | C | T | 137 | Ala |  | Ala | Ala |
| 412 | TTA |  | ATT | TTA | 138 | Leu |  | Ile | Leu |
| 450 | T |  | C | T | 150 | Gly | Gly | Gly | Gly |
| 577 | A |  | G | A | 193 | Ile |  | Val | Ile |
| 583 | T | C | C | T | 195 | Leu | Leu | Leu | Leu |
| 600 | G |  | A | G | 200 | Leu | Leu | Leu | Leu |
| 607 | A |  | T | A | 203 | Thr |  | Ser | Thr |
| 642 | T | C | C | T | 214 | Cys | Cys | Cys | Cys |
| 644 | A |  | G | A | 215 | Asn |  | Ser | Asn |
| 666 | T | C | C | T | 222 | Ser | Ser | Ser | Ser |
| 718 | T |  | G | T | 240 | Leu |  | Val | Leu |
| 722 | A |  | G | A | 241 | His |  | Arg | His |
| 761 | C |  | T | C | 254 | Thr |  | Ile | Thr |
| 765 | C |  | G | C | 255 | Ala |  | Ala | Ala |

Note: Rows shaded in yellow have non-synonymous divergent sites between the two reference genomes, but in at least one species the alternative allele matches the reference allele of the other species.
