# Supplementary material for: Regulatory changes in the fatty acid elongase eloF underlie the evolution of sex-specific pheromone profiles in Drosophila prolongata
Source: BMC Biol. 2025 Apr 30;23:117. doi: 10.1186/s12915-025-02220-z (PMC12044895; doi:10.1186/s12915-025-02220-z)
Supplement: Supplementary file 18 — Additional file 18: Table S6. qPCR analysis of GFP transcript expression driven by eloF “long” constructs (complete eloF locus including flanking regions). [file 12915_2025_2220_MOESM18_ESM.docx]

Table S6. High fidelity *honghaier* sequence occurrence

| Species | honghaier ORF (414bp) count | honghaier (894bp) count |
| --- | --- | --- |
| *D. prolongata* | 1942 | 274 |
| *D. carrolli* | 1617 | 363 |
| *D. rhopaloa* | 3432 | 854 |
| *D. kurseongensis* | 2180 | 310 |
| *D. fuyamai* | 1742 | 11 |
| *D. elegans* | 0 | 0 |
| D. melanogaster | 0 | 0 |

Note: using BLASTn 2.2.31+. The cutoffs for *honghaier* high fidelity homologs are >90% query cover and >90% percent identity.
