# Supplementary material for: Regulatory changes in the fatty acid elongase eloF underlie the evolution of sex-specific pheromone profiles in Drosophila prolongata
Source: BMC Biol. 2025 Apr 30;23:117. doi: 10.1186/s12915-025-02220-z (PMC12044895; doi:10.1186/s12915-025-02220-z)
Supplement: Supplementary file 20 — Additional file 20: Table S7. RNA-seq data summary. [file 12915_2025_2220_MOESM20_ESM.docx]

Table S7. RNA-seq data summary.

| **Sample** | **Raw Reads** | **Clean Reads** | **Effective Rate (%)** | **Error Rate (%)** | **Q20(%)** | **Q30(%)** | **GC Content (%)** |
| --- | --- | --- | --- | --- | --- | --- | --- |
| Dpro_F_1 | 30344414 | 18193778 | 59.96 | 0.01 | 98.16 | 95.34 | 55.05 |
| Dpro_F_2 | 25460124 | 15361829 | 60.34 | 0.01 | 98.06 | 95.12 | 54.3 |
| Dpro_F_3 | 36316550 | 19245326 | 52.99 | 0.01 | 98.32 | 95.69 | 53.77 |
| Dpro_F_4 | 37396183 | 19525669 | 52.21 | 0.01 | 98.18 | 95.44 | 54.78 |
| Dpro_M_1 | 26796536 | 16587626 | 61.9 | 0.01 | 98.16 | 95.34 | 53.14 |
| Dpro_M_2 | 28896516 | 18187156 | 62.94 | 0.01 | 97.93 | 94.87 | 53.13 |
| Dpro_M_3 | 25315750 | 13188023 | 52.09 | 0.01 | 98.09 | 95.31 | 52.68 |
| Dpro_M_5 | 7206195 | 3294602 | 45.72 | 0.01 | 98.3 | 95.66 | 51.72 |
| Dcar_F_1 | 29174061 | 15503044 | 53.14 | 0.01 | 98.07 | 95.23 | 52.38 |
| Dcar_F_2 | 28112240 | 15288035 | 54.38 | 0.01 | 98.17 | 95.4 | 53.78 |
| Dcar_F_4 | 36082291 | 21015328 | 58.24 | 0.01 | 98.38 | 95.84 | 51.09 |
| Dcar_F_6 | 35006571 | 19051828 | 54.42 | 0.01 | 97.97 | 95.05 | 53.41 |
| Dcar_M_1 | 22562361 | 13618258 | 60.36 | 0.01 | 98.24 | 95.53 | 53.19 |
| Dcar_M_2 | 27694508 | 15030603 | 54.27 | 0.01 | 98.25 | 95.58 | 52.69 |
| Dcar_M_3 | 25947915 | 15636694 | 60.26 | 0.01 | 98.14 | 95.34 | 51.78 |
| Dcar_M_5 | 21910989 | 11529155 | 52.62 | 0.01 | 98.15 | 95.39 | 51.62 |

Raw reads: the total amount of reads of raw data (read1 + read2), every four lines taken as one unit.

Clean reads: the total amount of reads of clean data, each of four lines taken as one unit.

Effective Rate (%): (Clean reads/Raw reads) * 100%

Error rate: base error rate

Q20, Q30: (Base count of Phred value > 20 or 30) / (Total base count)

GC content: (G & C base count) / (Total base count)
