# Supplementary material for: Regulatory changes in the fatty acid elongase eloF underlie the evolution of sex-specific pheromone profiles in Drosophila prolongata
Source: BMC Biol. 2025 Apr 30;23:117. doi: 10.1186/s12915-025-02220-z (PMC12044895; doi:10.1186/s12915-025-02220-z)
Supplement: Supplementary file 21 — Additional file 21: Table S8. RNA-seq read mapping statistics. [file 12915_2025_2220_MOESM21_ESM.docx]

Table S8. RNA-seq read mapping statistics.

| **Sample** | **M Aligned (% Aligned)** | **Overlapping Genes** | **No Feature** | **Ambiguous Features** | **Multi**  **mapping** | **Unmapped** |
| --- | --- | --- | --- | --- | --- | --- |
| Dpro_F_1 | 12.2 (92.8%) | 11112851 (84.3%) | 797692 (6.1%) | 326756 (2.5%) | 796396 (6.0%) | 147450 (1.1%) |
| Dpro_F_2 | 10.9 (92.9%) | 9764869 (83.1%) | 863405 (7.3%) | 288170 (2.5%) | 709311 (6.0%) | 124662 (1.1%) |
| Dpro_F_3 | 13.2 (89.5%) | 11762091 (80.0%) | 1052235 (7.2%) | 348850 (2.4%) | 922849 (6.3%) | 618591 (4.2%) |
| Dpro_F_4 | 12.8 (86.9%) | 11607811 (78.6%) | 876462 (5.9%) | 352797 (2.4%) | 961721 (6.5%) | 974142 (6.6%) |
| Dpro_M_1 | 12.7 (92.3%) | 11382212 (82.9%) | 882537 (6.4%) | 416154 (3.0%) | 909163 (6.6%) | 145253 (1.1%) |
| Dpro_M_2 | 12.9 (92.2%) | 11595790 (83.2%) | 849273 (6.1%) | 407331 (2.9%) | 924248 (6.6%) | 167338 (1.2%) |
| Dpro_M_3 | 11.3 (85.8%) | 10172545 (76.9%) | 812473 (6.1%) | 354630 (2.7%) | 848611 (6.4%) | 1033239 (7.8%) |
| Dpro_M_5 | 3.9 (89.3%) | 3468903 (79.9%) | 268893 (6.2%) | 137750 (3.2%) | 348931 (8.0%) | 114818 (2.6%) |
| Dcar_F_1 | 13.2 (95.5%) | 12195693 (88.0%) | 663784 (4.8%) | 375294 (2.7%) | 460058 (3.3%) | 166504 (1.2%) |
| Dcar_F_2 | 13.4 (96.4%) | 12414444 (89.5%) | 603605 (4.4%) | 349775 (2.5%) | 367662 (2.7%) | 132232 (1.0%) |
| Dcar_F_4 | 12.9 (95.7%) | 11847580 (87.8%) | 670845 (5.0%) | 385196 (2.9%) | 438529 (3.3%) | 148504 (1.1%) |
| Dcar_F_6 | 14.4 (91.2%) | 13315646 (84.0%) | 738847 (4.7%) | 395172 (2.5%) | 441754 (2.8%) | 951881 (6.0%) |
| Dcar_M_1 | 12 (96.2%) | 11039955 (88.6%) | 514678 (4.1%) | 435143 (3.5%) | 362917 (2.9%) | 110586 (0.9%) |
| Dcar_M_2 | 13 (94.0%) | 11949586 (86.2%) | 636828 (4.6%) | 448819 (3.2%) | 367462 (2.7%) | 459323 (3.3%) |
| Dcar_M_3 | 12.6 (96.2%) | 11563218 (88.6%) | 591867 (4.5%) | 401992 (3.1%) | 373925 (2.9%) | 124977 (1.0%) |
| Dcar_M_5 | 10.2 (91.3%) | 9297876 (83.3%) | 580338 (5.2%) | 307213 (2.8%) | 348561 (3.1%) | 624109 (5.6%) |

Ambiguous features: reads that overlap with two or more features

Multi mapping: reads that map to more than one location in the genome
