# Supplementary material for: Regulatory changes in the fatty acid elongase eloF underlie the evolution of sex-specific pheromone profiles in Drosophila prolongata
Source: BMC Biol. 2025 Apr 30;23:117. doi: 10.1186/s12915-025-02220-z (PMC12044895; doi:10.1186/s12915-025-02220-z)
Supplement: Supplementary file 30 — Additional file 30: Table S9. Primers used for cloning and qPCR. [file 12915_2025_2220_MOESM30_ESM.docx]

Table S9. Primers used for cloning and qPCR.

| Purpose | Primer name | Primer sequence (5' -> 3') | Note |
| --- | --- | --- | --- |
| pCR8::  Dcar eloF WT^(l)^ + TE | KB86_eloF_full_pCR8_F | CAGGCTCCGAATTCGCCCTTGTGTACCTTCTTATCCACAGCT |  |
|  | KB86_eloF_noTE_pCR8_R | GGATGCTAAATTAAAAAGTTCCTTAAAAGCTGAACT |  |
|  | Bavi_eloF_TE_pCR8_F | GGAACTTTTTAATTTAGCATCCATGTTTTAAGT |  |
|  | Bavi_eloF_full_pCR8_R | GCTGGGTCGAATTCGCCCTTCGAAGTTGATATACCCTTGCA |  |
| pCR8::  Dpro eloF WT^(l)^ - TE | Bavi_eloF_full_pCR8_F | CAGGCTCCGAATTCGCCCTTGGGTACCTTCTTAACCACAGCT |  |
|  | Bavi_eloF_noTE_pCR8_R | TGGGTCGAATTCGCCCTTTAAAAGGTTCCTTAAAAGCTGAACT |  |
| pCR8::  Dpro eloF WT^(l)^ | Bavi_eloF_full_pCR8_F | CAGGCTCCGAATTCGCCCTTGGGTACCTTCTTAACCACAGCT |  |
|  | Bavi_eloF_full_pCR8_R | GCTGGGTCGAATTCGCCCTTCGAAGTTGATATACCCTTGCA |  |
| pCR8::  Dcar eloF WT^(l)^ | KB86_eloF_full_pCR8_F | CAGGCTCCGAATTCGCCCTTGTGTACCTTCTTATCCACAGCT |  |
|  | KB86_eloF_full_pCR8_R | GCTGGGTCGAATTCGCCCTTTAAAAAGTTCCTTAAAAGCTGAACT | |
| pCR8::  Dpro eloF WT^(s)^ | Bavi_eloF_down_pCR8_F | GGCTCCGAATTCGCCCTTCCAATTGGTGTGCTTTAAGACT |  |
|  | Bavi_eloF_down_pCR8_R | TGGGTCGAATTCGCCCTTCGAAGTTGATATACCCTTGCA |  |
| pCR8::  Dpro eloF WT^(s)^ - TE | Bavi_eloF_down_pCR8_F | GGCTCCGAATTCGCCCTTCCAATTGGTGTGCTTTAAGACT |  |
|  | Bavi_eloF_noTE_pCR8_R | TGGGTCGAATTCGCCCTTTAAAAGGTTCCTTAAAAGCTGAACT |  |
| pCR8::  Dcar eloF WT^(s)^ | KB86_eloF_down_pCR8_F | GGCTCCGAATTCGCCCTTCCAAGTGGTGTGCTTTAAGGC |  |
|  | KB86_eloF_down_pCR8_R | TGGGTCGAATTCGCCCTTTAAAAAGTTCCTTAAAAGCTGAACT |  |
| pCR8::  Dcar eloF WT^(s)^ + TE | Bavi_eloF_TE_pCR8_F | GGAACTTTTTAATTTAGCATCCATGTTTTAAGT |  |
|  | Bavi_eloF_down_pCR8_R | TGGGTCGAATTCGCCCTTCGAAGTTGATATACCCTTGCA |  |
|  | KB86_eloF_down_pCR8_F | GGCTCCGAATTCGCCCTTCCAAGTGGTGTGCTTTAAGGC |  |
|  | KB86_eloF_noTE_pCR8_R | GGATGCTAAATTAAAAAGTTCCTTAAAAGCTGAACT |  |
| Sanger sequencing (pCR8 plasmid) | M-13F (-20) | GTAAAACGACGGCCAGT |  |
|  | M13-R (-26) | CAGGAAACAGCTATGAC |  |
| Sanger sequencing (pGreenFriend plasmid) | pGF_seqF0 | AAATAGGGGTTCCGCGCACAT |  |
|  | EGFP-N | CGTCGCCGTCCAGCTCGACCAG |  |
| Sanger sequencing (insert) | eloF_F2 | AACGCTGTGATGTTGGTATTGG |  |
|  | eloF_R2 | CTCCCTATCCTTGTGCTCGTG |  |
|  | eloF_F1 | AGAGGAGTTTGTGGTGGAAGAAGT |  |
|  | eloF_R1 | CGTAAAGGAGGCAGTCAGAAGTG |  |
|  | eloF_down_F4 | AAAAGATTCCCCATTCAAAAACTGA |  |
|  | eloF_down_F3 | GGTGTGTGCAAATTTTCAATTCGAT |  |
|  | eloF_down_F2 | ATATGGCATCTACAGGATATAGCCG |  |
| qPCR | eloF_F5 | CTTTTGATCTGCGTGGCGTTA | R^2^=0.9973, efficiency=95.8% |
|  | eloF_R5 | CAGGAAGTGTGCTCCCAATAC | reside on two exons |
|  | eGFP_F1 | CCACATGAAGCAGCACGACTT | R^2^=0.9936, efficiency=104.1% |
|  | eGFP_R1 | CGTGCGCTCCTGGACGTA |  |
|  | Rpl32_F1 | ATGCTAAGCTGTCGCACAAATG | reside on two exons |
|  | Rpl32_R1 | GTTCGATCCGTAACCGATGT | R^2^=0.9984, efficiency=99.2% |
